# Effect of Meal Ingestion on Liver Stiffness in Patients with Cirrhosis and Portal Hypertension

**DOI:** 10.1371/journal.pone.0058742

**Published:** 2013-03-08

**Authors:** Annalisa Berzigotti, Andrea De Gottardi, Ranka Vukotic, Sith Siramolpiwat, Juan G. Abraldes, Juan Carlos García-Pagan, Jaime Bosch

**Affiliations:** 1 Hepatic Hemodynamic Laboratory, Liver Unit, Hospital Clinic, Institut d'Investigacions Biomediques August Pi i Sunyer (IDIBAPS), Barcelona, Spain; 2 Centro de Investigación Biomédica en Red de Enfermedades Hepáticas y Digestivas (CIBERehd), University of Barcelona, Barcelona, Spain; CIMA. University of Navarra, Spain

## Abstract

**Background and Aims:**

Liver stiffness is increasingly used in the non-invasive evaluation of chronic liver diseases. Liver stiffness correlates with hepatic venous pressure gradient (HVPG) in patients with cirrhosis and holds prognostic value in this population. Hence, accuracy in its measurement is needed. Several factors independent of fibrosis influence liver stiffness, but there is insufficient information on whether meal ingestion modifies liver stiffness in cirrhosis. We investigated the changes in liver stiffness occurring after the ingestion of a liquid standard test meal in this population.

**Methods:**

In 19 patients with cirrhosis and esophageal varices (9 alcoholic, 9 HCV-related, 1 NASH; Child score 6.9±1.8), liver stiffness (transient elastography), portal blood flow (PBF) and hepatic artery blood flow (HABF) (Doppler-Ultrasound) were measured before and 30 minutes after receiving a standard mixed liquid meal. In 10 the HVPG changes were also measured.

**Results:**

Post-prandial hyperemia was accompanied by a marked increase in liver stiffness (+27±33%; p<0.0001). Changes in liver stiffness did not correlate with PBF changes, but directly correlated with HABF changes (r = 0.658; p = 0.002). After the meal, those patients showing a decrease in HABF (n = 13) had a less marked increase of liver stiffness as compared to patients in whom HABF increased (n = 6; +12±21% vs. +62±29%,p<0.0001). As expected, post-prandial hyperemia was associated with an increase in HVPG (n = 10; +26±13%, p = 0.003), but changes in liver stiffness did not correlate with HVPG changes.

**Conclusions:**

Liver stiffness increases markedly after a liquid test meal in patients with cirrhosis, suggesting that its measurement should be performed in standardized fasting conditions. The hepatic artery buffer response appears an important factor modulating postprandial changes of liver stiffness. The post-prandial increase in HVPG cannot be predicted by changes in liver stiffness.

## Introduction

Liver stiffness (LS) measurement by transient elastography is a non-invasive ultrasound-based technique increasingly used to estimate the degree of fibrosis in patients with chronic liver diseases [1]. Since liver fibrosis is the major factor contributing to portal hypertension [2], LS is able to predict the presence of portal hypertension in patients with cirrhosis [2]. Interestingly, in this population increasing values of LS are associated [3] and predict [4] clinical end-points such as portal hypertension-related complications and hepatocellular carcinoma [5]. Hence, it is necessary to avoid under- or overestimation of LS in cirrhosis since this would lead to erroneous conclusions about the patients’ clinical risk.

On the other hand LS increases transiently and independently of fibrosis in a number of pathological conditions, including cholestasis [6], hepatic inflammation [7] and liver congestion [8], [9], and measurement standardization is a major issue.

Meal ingestion causes a physiological increase of splanchnic blood flow, which in patients with cirrhosis is associated with an acute increase in HVPG[10]–[13]. It has been proposed that meals may influence LS in patients with mild chronic hepatitis [14], but there is no information on the post-prandial variation of LS in patients with cirrhosis, who represent the population at risk of clinical events.

In the present study we aimed at investigating whether a test meal induces changes in LS in patients with cirrhosis and whether these changes are influenced by the post-prandial increase occurring in portal blood flow and HVPG.

## Patients and Methods

### Patients

This study included 19 patients (14 men and 5 women) with cirrhosis and portal hypertension. The study was performed according to the principles of the Declaration of Helsinki, (revision of Edinburgh 2000) and was approved by the Human Research Ethics Committee of the Hospital Clinic (Comité Ético de Investigación Clínica, CEIC), Barcelona, Spain. The nature of the study was explained to the patients, and written informed consent was obtained in each case.

All patients had cirrhosis diagnosed by clinical, biochemical, ultrasonographic, and/or histological criteria. Table 1 summarizes the clinical features of the studied patients. Patients with alcoholic cirrhosis were abstinent from at least 6 months at the moment of the study.

**Table 1:** Main clinical and biochemical characteristics of patients included in the study (n = 19).

Parameter	Values	Values in patients in whom HVPG was measured (n = 10)
**Age (yrs)**	56±10	60±8
**Gender (M/F)**	14/5	5/5
**Etiology (HCV/alcohol/NASH)**	9/9/1	4/5/1
**Body Mass Index (Kg/m^2^)**	26.4±2.6	27.5±2.4
**Child-Pugh score**	6.9±1.8	7.0±1.9
**Child-Pugh class (A/B/C)**	10/7/2	6/2/2
**MELD score**	11±4	12±5
**Esophageal varices size (small/large)**	6/13	1/9
**Ascites (n)**	2	2
**Hepatic Encephalopathy (n)**	0	0
**Previous decompensation (n)**	12	7
**Receiving non-selective beta-blockers (n)**	13	7
**Dose of NSBB (mg/day)**	66±39	65±34
**Receiving Furosemide (n)**	2	1
**Receiving Aldosterone (n)**	3	1
**Albumin (g/dl)**	3.5±0.5	3.4±0.6
**INR**	1.38±0.27	1.43±0.34
**Bilirubin (mg/dl)**	1.4±0.3	2.1±1.3
**Creatinine (mg/dl)**	0.77±0.17	0.70±0.17
**ALT (U/l)**	44±27	38±18

NSBB: non-selective beta-blockers.

As shown, the mean Child-Pugh score was 6.9±1.8 points (range 5–10); all patients had esophageal varices, which were small in 6 cases, medium in 10, and large in 3.

12 patients had had previous decompensation of cirrhosis, and 7 were compensated. 2 patients had ascites at the moment of the study, which was minimal and did not preclude LS measurement. Mean body weight was 72±10 Kg.

Exclusion criteria were the following: obesity (BMI>30 Kg/m^2^), technical failure of transient elastography or Doppler-ultrasound and previous derivative treatment of portal hypertension (surgery or transjugular intrahepatic porto-systemic shunt).

### Portal Blood Flow, Hepatic Artery Blood Flow and Total Hepatic Blood Flow by Doppler US

The patients were transferred to the Hepatic Hemodynamics Laboratory in the morning after fasting for at least 6 h, and were let supine for at least 10 minutes before the baseline assessment. Doppler-ultrasound studies were performed with a Siemens ACUSON Sequoia™ 512 (Acuson, Mountain View, CA, USA) using a 3.75 multifrequency sector probe provided with pulsed, colour and power device, by a single trained operator.

The examination included a standard B-mode scan for the assessment of the diameter of portal vein (PV) and hepatic artery (HA), and a colour and pulsed Doppler examination for the assessment of PV and HA blood velocity. All these parameters were measured in standardized sites of the vessels, as previously reported[15]–[17]. Portal blood flow (PBF) was calculated as follows:
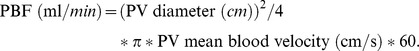



Hepatic artery blood flow (HABF) was similarly calculated.

Total hepatic blood flow (THBF) by Doppler was calculated as the sum of PBF and HABF. The hepatic artery buffer response was defined as a decrease of HABF of at least 10% from the baseline value after the test meal.

### Liver Stiffness by Transient Elastography

After Doppler-US assessment, LS was evaluated by transient elastography (Fibroscan®; Echosens, Paris, France). As previously described [2], measurements of LS were performed by the same experienced operator on the right lobe of the liver through intercostal spaces on patients lying in the dorsal decubitus position with the right arm in maximal abduction. The tip of the probe transducer was placed on the skin between the ribs at the level of the right hepatic lobe. The operator, assisted by an ultrasonic time-motion image, located a liver portion of at least 6 cm thick free of large vascular structures. 10 successful measurements were performed on each patient. Success rate was calculated as the ratio of the number of successful measurements over the total number of acquisitions. Only liver stiffness measurements with a success rate of at least 60% and an interquartile range lower than 30% were considered reliable. The results are expressed in kilopascal (kPa) and median value was kept as representative of liver stiffness. The whole examination duration was less than 5 minutes. To ensure a better reproducibility, the site of baseline measurement was pointed out by a marker-pen on the patient’s skin.

### Test Meal

After baseline examination all patients ingested the liquid test meal, that consisted in a milkshake in the iso-volumetric quantity of 7 ml/Kg of body weight containing both whole milk and sugar (0.08 g of proteins/ml, 0.32 g of carbohydrates/ml, and 0.26 g of lipids/ml for a total of 4 kcal/Kg) and 1 unit of Ensure Plus (237 ml, Abbott Netherlands, containing 13 g of proteins, 50 g of carbohydrates, and 11 g of lipids for a total of 350 kcal). Thus, for a 75 Kg individual, the test meal provided 650 kcal, equivalent to the caloric intake of a continental breakfast or a regular Mediterranean lunch. The test meal was administered in 5 minutes. After 30 minutes of the end of meal ingestion all the above mentioned non-invasive measurements were repeated.

### HVPG Measurement

In 10 patients the HVPG response to the standard meal was also assessed. Immediately after baseline ultrasound examination and LS measurement patients underwent hepatic vein catheterisation. Under local anaesthesia, with ultrasonographic guidance (SonoSite Inc, Bothell, WA) a 8F venous catheter introducer (Axcess; Maxxim Medical, Athens, TX, USA) was placed in the right internal jugular vein using the Seldinger technique. Thereafter, a 7F balloon-tipped catheter (Edwards Lifesciences, Irvine, CA, USA) was advanced into the right hepatic vein to measure wedged and free hepatic venous pressures (WHVP and FHVP, respectively) by the connection to external electro-mechanical transducer and polygraph (Mac-Lab®, GE Healthcare, Freiburg, Germany). Hepatic venous pressure gradient (HVPG) was calculated as the difference between wedged and free hepatic venous pressure, as previously described [18]. HVPG measurement was performed at baseline and after 30 minutes of standard liquid meal ingestion; all measurements were performed in triplicate, and permanent tracings were recorded.

#### Statistical analysis

The comparison between baseline and post-prandial measurements in the study population was assessed by Wilcoxon’s test. Comparisons between different groups were performed by Mann-Whitney’s U test. Correlations were assessed by Spearman’s r test.

The α value was set at 0.05. All p-values are two-sided. Statistical analysis was performed with SPSS 16.0 package (SPSS, Chicago, IL, USA).

## Results

The baseline and post-prandial values of the studied parameters are shown in Table 2.

**Table 2:** Hemodynamic data at baseline and after the standard meal in the entire studied population (n = 19).

	Baseline	After the standard meal	Change (%)	p
**Liver stiffness (kPa)**	40.7±16.9	51.2±21.0	27.5±33.3	<0.0001
**PBF (ml/min)**	893±554	1127±536	33.0±30.9	<0.0001
**HABF (ml/min)**	278±247	171±116	−20.2±45.4	0.04
**HABF (% of THBF)**	24.5±14.4	13.9±8.5	−28.8±43.3	0.12
**THBF (ml/min)**	1171±562	1298±571	13.9±19.3	0.03
**HVPG (mmHg) (n = 10)**	16.4±4.7	20.2±3.9	26.4±13.4	0.003

Data are shown as mean±SD. p values refer to the comparison between baseline and post-prandial values.

### Liver Stiffness Changes after the Standard Meal

**Figure 1:**
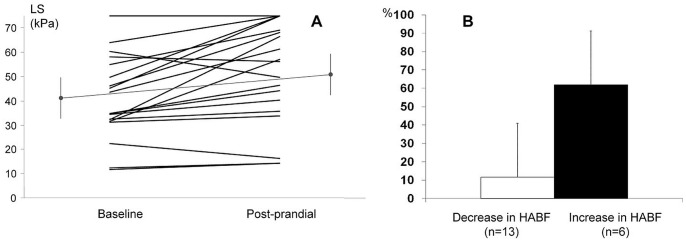
Changes in liver stiffness (LS) after a liquid test meal in the studied population of patients with cirrhosis (n = 19). Panel A. Individual changes of liver stiffness (LS) after the standard meal in the study population (n = 19). Baseline and post-prandial mean value (and standard deviation) are depicted in grey. **Panel B.** Changes in liver stiffness (LS) in patients showing a decrease in hepatic artery blood flow (HABF) post-prandially (column in white, n = 13) vs. HABF increased post-prandially (column in black, n = 6). As shown, patients showing a HABF decrease after the meal had a much lower increase of LS as compared to patients increasing HABF post-prandially (p<0.0001).

LS increased markedly post-prandially, by 27.5±33.3% (p<0.0001). LS increase was observed in 15 out of 19 patients; in the remaining four patients LS was unchanged in 1 and decreased in 3. Individual changes are shown in Figure 1, Panel A.

No differences in LS change were seen between patients with HCV-related cirrhosis vs. other etiologies, compensated vs. decompensated patients, and between patients with small varices vs. those with medium/large varices. LS increased more in the 13 patients on NSBB as compared to untreated patients, although this was not statistically significant (31.5±38.1% vs. 16.3±8.0%, p = 0.308).

### Post-prandial Changes of Portal Blood Flow, Hepatic Artery Blood Flow, Total Hepatic Blood Flow and HVPG

PBF increased markedly after the test meal (+33.0±30.9%, p<0.0001 vs. baseline); this increase was observed in all patients except one, in whom PBF did not change.

HABF showed overall a buffer response, decreasing by 20.2±45.4% (p = 0.04 vs. baseline). Nonetheless, HABF decreased in 13 patients (mean change −41.8±24.8%), while it increased in 6 cases (mean change +38.5±5.7%).

The fraction of liver perfusion contributed by the hepatic artery tended to decrease after the meal (−28.8±43.2%, p = 0.12). This was particularly evident in the subgroup exhibiting the expected hepatic artery buffer response (−52.8±20.2% vs. +23.2±63.1% in patients lacking the buffer response, p = 0.001).

As a consequence of the above mentioned results, THBF increased slightly in the whole population, by 13.9±19.3% (p = 0.03 vs. baseline).

In the 10 patients undergoing HVPG measurement (mean HVPG 16.4±4.7 mmHg) the post-prandial hyperemia was associated with a highly significant increase of HVPG (+26.4±13.4%, p = 0.003). In these patients liver stiffness increased similarly to the remaining part of the population (+31.4±32.2% vs. +23.2±34.8%, p = 0.497).

### Correlation among Post-prandial Changes of the Studied Parameters

LS changes did not correlate with changes in PBF (r = −0.27, p = 0.272), THBF (r = 0.16, p = 0.647) or HVPG (r = 0.32, p = 0.364).

Conversely, LS changes showed a direct and significant correlation with changes in HABF both in the whole population (r = 0.658; p = 0.002) and in the subgroup in which the HVPG was measured (r = 0.699; p = 0.013). Patients showing the expected decrease in HABF post-prandially (n = 13) had a significantly lower increase in LS as compared to patients in whom HABF increased post-prandially (n = 6) (+11.6±20.9% vs. +61.9±29.3%, p<0.0001) (Figure 1 Panel B).

## Discussion

LS is currently the best non-invasive surrogate predictor of portal hypertension in patients with cirrhosis [19], and its prognostic value was recently confirmed by cross-sectional studies about the presence of gastroesophageal varices [3], [20], [21] and longitudinal studies with hard clinical end-points such as the appearance of clinical decompensation and hepatocellular carcinoma [4], [5]. Since LS measurement is increasingly used in clinical practice in patients with cirrhosis as a help for prognostic stratification, physicians should be aware of factors causing misleading LS values.

In the present study we report that LS increases markedly after a meal in patients with cirrhosis and portal hypertension (by over 25%). This observation carries an important practical message, since it implies that LS should be always measured in fasting conditions to guarantee reliable values and avoid overestimation in this population.

Our results differ from those reported in the study by Mederacke et al. [14] since, contrarily to what we observed, in their study patients with cirrhosis (baseline values >10 kPa) LS did not increase after a standard meal (600 kcal). All our patients belong to this category, but we observed a marked post-prandial increase of LS. This discrepancy might be due to different factors. First, in our study the timing of the measurements was set at 30 minutes after the test meal, while it was immediately after and 1 hour after the standard meal in the study by Mederacke et al. [14]; our time-point was chosen according to previous observations by our group and others indicating that post-prandial hyperaemia and the post-prandial increase of portal pressure are maximum 30 minutes after meal ingestion [10]. The lack of later time-points is a limitation of the present study that makes difficult to specify for how long the patients should fast before TE. In this regard, it is interesting to note that a ultrasound study on the circadian variation of portal blood flow (PBF) in cirrhosis showed that the hyperaemic response is greater after the morning meal, and that PBF returns to pre-meal values only after circa 4 hours [22]. Therefore, we believe that similarly to what recommended for ultrasound, TE should be performed preferably in the morning after overnight fast or in any case after at least 4 hours fast, until new studies specifically address this issue.

Another potential limitation is that we did not assess in this study the intra-observer variability of Doppler-US and TE measurements. In this regard, previous studies suggest that both inter- and intraobserver variability are low for TE [23]. As for Doppler-US, intraobserver variability is low for experienced operators, as it was in the case of this study, and even inter-observer variability is low if measurements are taken according to standardized protocols [15], [24].

Our patients had a severe liver disease, showing established portal hypertension and esophageal varices. We chose studying this homogeneous population since in this setting the effects of meals on splanchnic hemodynamics are well characterized [10], [25] and since the presence and extent of collaterals influence the hemodynamic response to a meal [10], [25].

As for the relationship between the postprandial increase in portal pressure [10] and LS, we acknowledge that the number of patients studied is small and does not allow driving definite conclusions. With these limits, the lack of correlation between the post-prandial increase in HVPG and the postprandial increase in LS suggests that changes in LS do not mirror the increase in portal pressure, and that factors other than increased PBF and HVPG might modulate the post-prandial increase in LS.

In this regard, we observed that in patients with efficient post-prandial buffer response of the hepatic artery blood flow (physiologic response to increased portal blood flow after a meal) the LS increase was significantly less marked. As a consequence those patients lacking this physiologic adaptative mechanism had a much greater increase of LS, probably reflecting a greater contribution of the hepatic arterial blood flow in the total liver perfusion.

Finally, 13/19 patients were receiving propranolol at the moment of the present study. Since propranolol does not blunt postprandial hyperemia [26] and the consequent post-prandial increase in portal pressure [10], it is not surprising that TE increase occurred similarly (and even slightly more pronounced) in patients treated with NSBB.

In conclusion, standard liquid meal ingestion is associated with a marked increase of LS in patients with cirrhosis, suggesting that measurement of LS should be always performed in fasting conditions in this population. LS increase is more pronounced in patients lacking the post-prandial buffer response of hepatic artery blood flow, which appears to be an important factor modulating post-prandial changes in LS.
